# Observation of Extrinsic Photo-Induced Inverse Spin Hall Effect in a GaAs/AlGaAs Two-Dimensional Electron Gas

**DOI:** 10.1186/s11671-018-2715-y

**Published:** 2018-10-11

**Authors:** Jinling Yu, Xiaolin Zeng, Yumeng Wang, Lijia Xia, Shuying Cheng, Yonghai Chen, Yu Liu, Yunfeng Lai, Qiao Zheng

**Affiliations:** 1 Institute of Micro/Nano Devices and Solar Cells, School of Physics and Information Engineering, Fuzhou University, Fuzhou, China; 2 Key Laboratory of Semiconductor Materials Science, Institute of Semiconductors, Chinese Academy of Sciences, Beijing, 100083 China; 3 College of Materials Science and Opto-Electronic Technology, University of Chinese Academy of Sciences, Beijing, 100049 China; 4 Jiangsu Collaborative Innovation Center of Photovolatic Science and Engineering, Changzhou University, Changzhou, 213164 Jiangsu China

**Keywords:** Inverse spin Hall effect, Photo-induced inverse spin Hall current, Power dependence, Extrinsic mechanism, Light profile dependence

## Abstract

The inverse spin Hall effect induced by circularly polarized light has been observed in a GaAs/AlGaAs two-dimensional electron gas. The spin transverse force has been determined by fitting the photo-induced inverse spin Hall effect (PISHE) current to a theoretical model. The PISHE current is also measured at different light power and different light spot profiles, and all the measurement results are in good agreement with the theoretical calculations. We also measure the PISHE current at different temperatures (i.e., from 77 to 300 K). The temperature dependence of the PISHE current indicates that the extrinsic mechanism plays a dominant role, which is further confirmed by the weak dependence of the PISHE current on the crystal orientation of the sample.

## Background

Spintronics has attracted much attention due to its potential applications in information technology as well as revealing fundamental questions on the physics of electron spin in condensed matter [1–4]. The spin Hall effect (SHE) and its Onsager reciprocal, the inverse spin Hall effect (ISHE), play a significant role in spintronics since they provide an electrical method to convert charge current into spin current and vice versa, via spin-orbit coupling (SOC) [2, 5–8]. The SHE and ISHE have been widely studied in metallic films with heavy elements, such as Pt, Ta, Py, and IrMn, and the emerging topological insulators, such as Bi_2_Se_3_ and SnTe, due to their strong SOC [9–14]. These two effects are also observed in semiconductors, such as GaAs, ZnO, Si, Ge, GaN/AlGaN, and GaAs/AlGaAs two-dimensional electron gas [15–20].

The spin-to-charge current conversion in semiconductors is an important issue, since it open an avenue to integrate spintronics with electronics [5]. Photo-induced ISHE (PISHE) is recently emerging as an effective experimental tool to investigate the ISHE in semiconductors, which exploits a circularly polarized light with a Gaussian distribution to introduce a spin current into semiconductors and then utilizes the ISHE to generate a charge current [2, 19–22]. The PISHE current can be observed at room temperature, and it offers a convenient way to investigate the ISHE of semiconductors without introducing magnetic field and ferromagnetic elements [20]. Besides, the PISHE also paves a way to design new kinds of spin-photonics devices [22]. The PISHE current has been observed in GaN/AlGaN, GaAs/AlGaAs, and MgZnO/ZnO heterostructures [2, 19, 20]. However, the dependence of the PISHE current on the light power and light profile is still unknown.

There are two mechanisms for ISHE, i.e., intrinsic and extrinsic. The intrinsic mechanism is dependent only on the band structure of the perfectly order material [7, 23, 24], originating from Rashba [25–27] or Dresselhaus SOC [26], while the extrinsic mechanism refers to asymmetric Mott-skew or side-jump scattering from impurities in a spin-orbit coupled system [16, 24, 28, 29]. Although there are lots of studies investigating the intrinsic or extrinsic mechanism of ISHE, most of them are theoretical works, and very few experiential works focusing on this issue [16, 27, 30–32], because it is very difficult to distinguish these two mechanisms experimentally.

In this paper, we investigate the PISHE current in a GaAs/AlGaAs two-dimensional electron gas (2DEG). It is found that the PISHE current increases with increasing temperature, indicating that the PISHE current is mainly dominated by the extrinsic mechanism. This inference is further confirmed by the the weak dependence of the PISHE current on the crystal orientation of the sample. Besides, we also investigate the dependence of the PISHE current on the light power and light profile, which agrees very well with the theoretical model.

## Methods

The experiment is carried out on a (001)-oriented modulation-doped GaAs/AlGaAs 2DEG sample grown by molecular beam epitaxy (MBE) on a semi-insulating GaAs substrate. The electron density and the Hall mobility of the sample are measured to be 5.18 × 10^11^ cm ^−2^ and 3.97 × 10^3^ cm ^2^ *V*^−1^ *s*^−1^ at room temperature, respectively. The mobility of the 2DEG is a little low due to the background doping, which is in the order of 10^15^ or 10^16^ cm ^−3^, in the sample introduced during the sample growth. The sample is cleaved along [110] and $[1\bar {1}0]$ direction into a square of 10 × 10 mm^2^. Two pairs of ohmic contacts with a distance of 8 mm along [110] and [100] directions, respectively, are made by indium deposition and annealed at about 420 °C in nitrogen atmosphere.

**Fig. 1:**
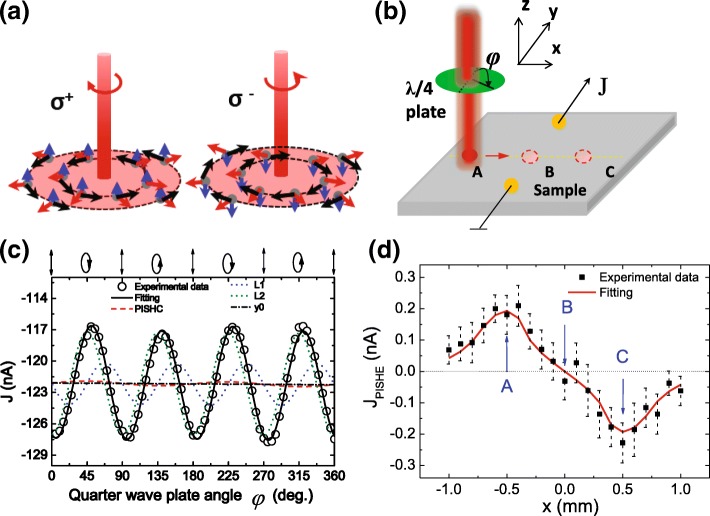
Method to obtain the PISHE current and the PISHE measured at 300 K. **a** Illustration of the movement of spin polarized electrons under the normal illumination of left-hand circular polarization (*σ*^+^) or right-hand circular polarization (*σ*^−^) light. The red arrows denote the flowing of electrons, the blue arrows indicate the spin direction of electrons, and the black arrows show the spin transverse force acting on electrons. **b** The geometry used to measure the PISHE current. **c** The photocurrent measured at 300 K as a function of the phase angle *φ* under normal incidence when the light spot is illustrated at point A. The solid line (black) is the fitting curve using Eq. (1), the dashed line (red) represents the PISHE current, and the blue and green dotted lines represent the *L*_1_ and *L*_2_ component. The dashed-dot line indicates the background current *J*_1_. **d** The PISHE current as a function of light spot locations measured at 300 K

A diode-pumped solid-state laser with a wavelength of 1064 nm is used as a radiation source. The laser beam passes through a chopper, a polarizer, and a quarter-wave plate and finally illuminates the sample vertically. Here, the polarizer and the rotating quarter-wave plate are used to change the light helicity *P*_*c*_= sin2*φ* from left-handed (*σ*^−^, *P*_*c*_=− 1) to right-handed (*σ*^+^, *P*_*c*_=+ 1) continuously, where *φ* is the angle between the polarization direction of the incident light and the optical axis of the quarter-wave plate. The light spot on the sample has a Gaussian profile. The current is collected between the two contacts along [100] (or [110]) direction of the sample by a preamplifier and a lock-in amplifier with a reference frequency of 229 Hz from the chopper. Figure 1a illustrates the setup used to measure the PISHE current.

For power-dependent measurements, the power of the light irradiated on the sample is changed from 250 to 40 mW by using attenuators. To change the profile of the light spot on the sample, optical lens with different focal distances is adopted. In the temperature-dependent measurements, the sample is mounted on an optical cryostat, which allows the variation of temperature from 77 to 300 K.

To obtain the relative ratio of Rashba to Dresselhaus SOC, we measure the photocurrent induced by circular photogalvanic effect (CPGE) for different crystallographic directions, i.e., the CPGE current is collected along [110] and [100] direction through the contacts, respectively, with the incident plane of light perpendicular to the connection of the two contacts. For the CPGE measurement, a similar experimental setup with that used in the PISHE measurement is adopted except that the light irradiates obliquely on the midpoint of the connection of the two contacts along [110] or [100] directions, and the angle of incidence ranges from − 40 to 40°. The CPGE current at a certain angle of incidence is extracted by fitting the light-polarization-state dependent photocurrent *J* collected along the two contacts to the following equation [33]: *J*=*J*_CPGE_ sin2*φ*+*L*_11_ sin4*φ*+*L*_22_ cos4*φ*+*J*_11_. Here, *J*_CPGE_ is the CPGE current, *L*_11_ and *L*_22_ are the photocurrent induced by linearly polarized light, and *J*_11_ is the background current originating from the photovoltaic effect or Dember effect [33].

## Results and Discussion

Under an illumination of a circularly polarized light with a Gaussian profile, spin polarized carriers with a Gaussian distribution in space will be generated in the absorption unsaturated area. As a result, a diffuse spin current appears which is flowing along the radial direction. Then, due to the ISHE effect, the spin polarized carriers experience a “spin transverse force” along the tangential direction, leading to a transverse charge current, i.e., a vortex current (named as PISHE current), in the axial direction [8, 20], as shown in Fig. 1a. As the polarization state of the light is changed from left-hand circular polarization (*σ*^+^) to the right-hand circular polarization (*σ*^−^), the spin polarization of electrons is changed from spin up to spin down, leading to the reversion of the spin transverse force and the PISHE current. As the quarter-wave plate rotates from 0 to 180°, i.e., as the angle *φ* is changed from 0 to 180°, the polarization state of the light is changed from vertically linear polarization (at 0°), to left-hand circular polarization (at 45°), vertically linear polarization (at 90 °), right-hand circular polarization (at 135°), and again vertically linear polarization (at 180°) sequently, as shown in the top part of Fig. 1c. Therefore, as the angle *φ* is changed from 45 to 135°, the PISHE is reversed, indicating that the PISHE is proportional to sin2*φ*. It is worth noting that, at a *φ* angle of 0, 90, and 180°, the light is linearly polarized. The linearly polarized light will also induce photocurrent due to the optical momentum alignment effect [34], named as *L*_1_, or due to the anisotropy optical absorption [35, 36], named as *L*_2_. The currents *L*_1_ and *L*_2_ induced by linearly polarized light are proportional to sin4*φ* and cos4*φ*, respectively. Besides, a background photocurrent *J*_1_ originating from the photovoltaic effect or Dember effect will also present, which is independent of the polarization state of the light. Thus, according to their different dependence on the angle *φ*, we can extract the PISHE current by fitting the experimentally measured light polarization state-dependent photocurrent *J* to the following formula [8, 33]:

1 $$ J=J_{\text{PISHE}}\sin 2\varphi+L_{1}\sin 4\varphi+L_{2} \cos 4\varphi+J_{1},  $$

where *J*_PISHE_ is the PISHE current excited by left-hand circular polarization light, *L*_1_ and *L*_2_ are the photocurrent induced by linearly polarized light, and *J*_1_ is the background current [19]. It should be noted that the *L*_2_ term has been included in the fitting equation, i.e., Eq. (1), due to the large optical anisotropy present in the sample. The optical anisotropy might be induced by anisotropic interface structures [37], segregation of atoms [38], or residual stress [39].

To obtain the spacial distribution of the PISHE, we sweep the the laser spot from the left to the right side of the two contacts along their perpendicular bisector [see Fig.1a]. At each spot position, we rotate the quarter-wave plate from 0 to 360° and obtain the PISHE current by fitting Eq. (1) to the experimentally measured light polarization state-dependent photocurrent *J*. Figure 1b shows a typical result of photocurrent measured as a function of the phase angle *φ*, when the laser spot is fixed at *x* =− 0.5 mm, i.e., at point A [see Fig. 1a]. The photocurrent is measured at 300 K and collected along the two contacts along [110] direction. The laser spot on the sample has a diameter of about 1.4 mm with a Gaussian profile and a power of 250 mW. The circles in Fig. 1b are the experimental data, and the solid line is the fitting result according to Eq. (1). It can be seen that the experimentally measured photocurrent fluctuates periodically with rotating the quarter-wave plate. This is because the photocurrent is a summation of the PISHE current, the photocurrent induced by linearly polarized light and the background current, and they show different dependence on angle *φ*. The dashed line indicates the PISHE current, and the dash-dotted line denotes the background current. The blue and green dotted lines represent the *L*_1_ and *L*_2_ component induced by linearly polarized light, respectively. One can see that the PISHE current is much smaller than that of the photocurrent induced by linearly polarized light.

The obtained PISHE current as a function of spot is shown in Fig. 1c. It can be seen that, as the laser spot is moved from left to the right side of the two contacts, the PISHE current reverses its direction. When the laser spot is focused on the midpoint of the two contacts, the PISHE current is almost equal to zero. This phenomenon can be quantitatively explained by a vortex current model induced by photo-induced inverse spin Hall effect [20]. Specifically, under the radiation of a laser with a Gaussian profile *G*(*r*)=$\frac {1}{\sqrt {2\pi }\sigma }\exp \left (-\frac {r^{2}}{2\sigma ^{2}}\right)$, a spin current flowing along the radial direction will be induced, which can be expressed as *j*_*r*_= *τ*_*s*_*D*∇_*r*_*G*(*r*). Here, *D* is the spin diffusion coefficient, *τ*_*s*_ is the spin relaxation time, *r* denotes the radial direction, and *σ* indicates the distribution variance related to the full width at half maximum (FWHM) of the light intensity. Due to the ISHE effect, the spin polarized carriers will experience a spin transverse force $f(r)\propto j_{r}\times \hat {z}$ [20, 40], which can be expressed as $f(r)=-f_{0}r/\sigma ^{3}\exp \left (-\frac {r^{2}}{2\sigma ^{2}}\right)$. Here, *f*_0_ is the spin transverse force constant associated with SOC of the material system. The vortex electric field $\vec {E}$ can be determined by the circular electromotive force (EMF), which can be written as $\varepsilon (r_{0})=\frac {2\pi }{q}\int _{0}^{r_{0}} f(r)rdr$, through $\oint \vec {E}(r_{0})\cdot d\vec {l}=\varepsilon (r_{0})$. Here, *r*_0_ is the radius of the light spot, and the integral loop is along the perimeter of the light spot. Therefore, we have

2 $$ \nabla\times \vec{E}(r_{0})=-\frac{f_{0}r}{q\sigma^{3}}\exp \left(-\frac{r^{2}}{2\sigma^{2}}\right).  $$

It is worth noting that the small difference between Eq. (2) and that reported in [20] is because the normalized Gaussian function is adopted in this paper, while the not-normalized Gaussian function was used in [20]. The *f*_0_ in this paper is equivalent to *f*_0_/*σ* reported in [20]. The electric current between the two contacts (named as *a* and *b*, respectively) can be expressed as

3 $$ {}I_{ab}\,=\,\frac{V_{ab}}{R_{ab}}\,=\,\frac{1}{R_{ab}}\!\int_{a}^{b}\! \vec{E}\cdot d\vec{l}\,=\,\frac{1}{R_{ab}}\!\oint_{abo}\!\vec{E}\cdot d\vec{l}\,=\,\frac{1}{R_{ab}}\iint_{S}\nabla\times\vec{E} ds,  $$

where *V*_*ab*_ (*R*_*ab*_) is the voltage (resistance) between the contacts *a* and *b*, *o* is the origin of the light spot, and *S* indicates the triangle area of *abo*. It should be mentioned that the absorption saturated area, in which the light intensity absorbed by the sample is a constant and it reaches the maximum absorption of the sample, should be deducted from the integral of Eq. (3). This is due to the fact that the gradient of the photo-generated carriers is zero in that area, and as a result, the spin current and the PISHE current are all zero in the area.

It is worth noting that Eq. (3) only holds when the contacts *a* and *b* are covered by the light spot, because outside the light spot Eq. (2) is no longer valid. Thus, taking into account the relation between the electric current outside (*J*_*f*_) and inside (*J*_*e*_) the spot, i.e., *J*_*f*_=$J_{e}\exp \left (-\frac {l}{A\cdot L_{s}}\right)$ [41], we can express Eq. (3) as:

4 $$ I_{ab}=\frac{1}{R_{ab}}\iint_{D}\nabla\times\vec{E}\cdot\exp \left(-\frac{l}{A\cdot L_{s}}\right)ds.  $$

Here, *l* is the distance between the edge of light spot and the connection of the two contacts, *L*_*s*_ is the diffusion length of electrons, and *A* is a constant. Using Eqs. (2) and (4) to fit the experimentally measured PISHE current, we can obtain the spin transverse force *f*_0_ and the diffusion length *A*·*L*_*s*_. The fitting result is shown in Fig. 1c by a solid line. One can see that the experimental data is well-fitted by the model. In the fitting, the following experimentally measured parameters are adopted, *σ* = 0.2 mm, *L* = 4 mm, *r*_0_ = 0.7 mm, and *R*_*ab*_ = 15.5 k *Ω*. The spin transverse force *f*_0_/*q* of electrons is fitted to be 6.8 × 10^−6^ N ·m/C at 300 K, *A*·*L*_*s*_ is fitted to be 2.8 × 10^−4^ m, and the radius of the absorption saturated area is fitted to be 0.34 mm, which indicates that the absorption saturated intensity of the light *I*_*c*_ corresponds to about one fifth of the maximum intensity *I*_*m*_, i.e., *I*_*c*_ = 1/5 *I*_*m*_.

**Fig. 2:**
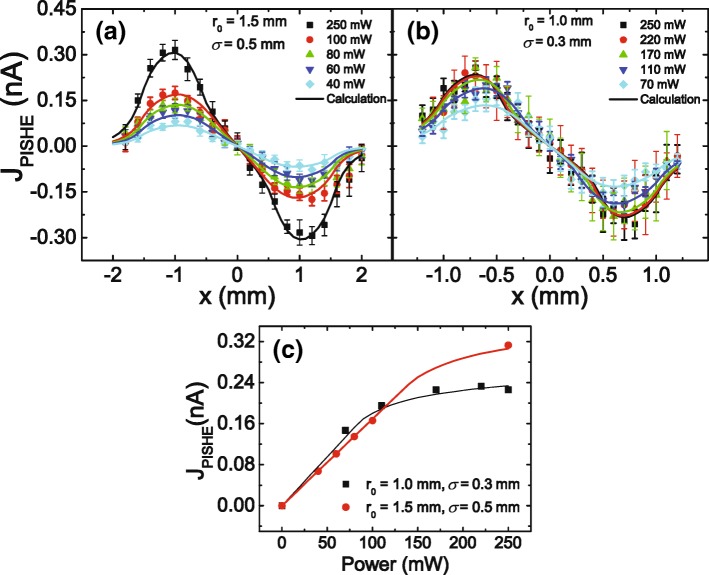
PISHE current as a function of light spot locations under different light power. **a**, **b** PISHE current excited by a Gaussian profile light spot with *r*_0_ = 1.5 mm and *σ* = 0.5 mm and *r*_0_ = 1 mm and *σ* = 0.3 mm, respectively. **c** The variation of the peak value of the PISHE current with the excitation power, in which the symbols and the solid lines are the experimental data and the theoretical calculation results, respectively

To investigate the dependence of the PISHE current on the light power and on the light profile, we perform the PISHE measurement under different light power and different light profiles. Figure 2a, b shows the PISHE current as a function of light spot locations under different light power with a light spot radius of *r*_0_ = 1.5 mm and *σ* = 0.5 mm and *r*_0_ = 1 mm and *σ* = 0.3 mm, respectively. The symbols are the experimental data, and the solid lines are the theoretical calculations according to Eqs. (2) and (4). In the calculations, the same parameters, except for the light spot parameters, adopted in Fig. 1c are used, i.e., *f*_0_/*q* = 6.8 × 10^−6^ N ·m/C, *A*·*L*_*s*_ = 2.8 × 10^−4^ m, *R*_*ab*_ = 15.5 k *Ω*, and *I*_*c*_ = 1/5 *I*_*m*_. Here, *I*_*m*_ is the maximum light intensity of light when the power is 250 mW. It can be seen that the intensity of the PISHE current increases with the light power, and under the power of 250 mW, the light spot with larger FWHM (i.e., larger *σ*) leads to larger PISHE current. We can also see that, for a light spot with larger FWHM, the peak of the PISHE curve will present at a larger value of *x*. Here, *x* is the distance between the light spot center and the midpoint of the connection of the two contacts. This is because the spin current and the resulting PISHE current are proportional to the gradient of the light profile. For a better comparison of the PISHE current induced by different light spot profiles, we summarize the results of Fig. 2a, b in Fig. 2c, i.e., we summarize the dependence of the peak value of the PISHE current on the excitation power for different light spot profiles in Fig. 2c, where the symbols indicate the experimental data, and the solid lines are the theoretical calculation results. One can see that the experimental results agree very well with the theoretical simulations, which confirms the model.

**Fig. 3:**
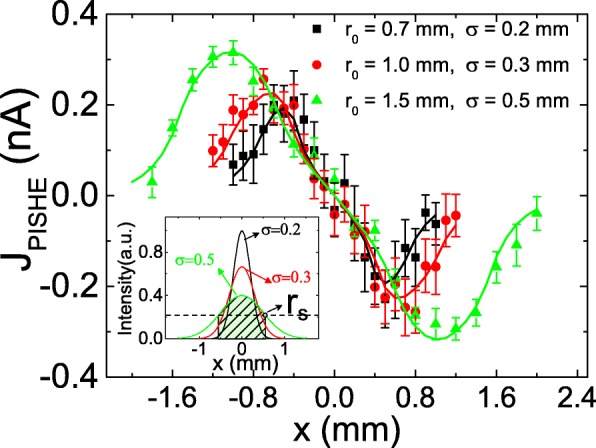
PISHE current as a function of light spot location under different Gaussian light profiles when the light power is 250 mW. The inset shows the distribution of light intensity for different Gaussian light profiles. The dashed line indicates the absorption saturation intensity of the sample

Figure 2c indicates that, as power increases, the PISHE current increases monotonically first and then becomes saturation gradually. The emergence of the saturation of the PISHE current with light power is due to the presence of absorption saturation at high power. When the maximum light intensity is smaller than the absorption saturation intensity, the PISHE current increases linearly with the light power. When the maximum light intensity is larger than the absorption saturation intensity, the PISHE current is tending to saturation with increasing light power. The influence of the light spot size on the PISHE current can be understood in the aspect of the affect of FWHM of the light spot on the PISHE current, because the light spot size and the FWHM are correlated with each other by light power. Specifically, for a certain light power, a larger size of light spot has a larger value of FWHM. At a certain light power, if the maximum light intensity is smaller than the absorption saturation intensity, a light profile with a smaller FWHM (i.e., smaller size of light spot) can generate larger PISHE current, because a smaller FWHM will result in a larger spin current; while if the maximum light intensity is larger than the absorption saturation intensity, a light profile with a smaller value of FWHM will lead to smaller PISHE current. This can also be clearly seen in Fig. 3, which summarizes the PISHE current as a function of light spot locations under different light profiles. The power of the light is 250 mW. It can be seen that, as the value of *σ* increases from 0.2 to 0.5 mm, the peak value of PISHE current decreases monotonously. This is because in the absorption saturated area, there is no spin current, and as a result, no PISHE current is generated. Therefore, the light inside the absorption saturated area does not make any contribution to the PISHE current. The inset of Fig. 3 shows the distribution of light intensity for different Gaussian light profiles. The dashed line represents the absorption saturation intensity of the sample. The intersection points between the dashed line and the light intensity curves indicate the radius of the absorption saturated areas, denoted as *r*_*s*_. The light within the circular area of radius *r*_*s*_, which is indicated by the shadow area when *r*_0_ = 1.5 and *σ* = 0.5 mm, does not contribute to the PISHE current. It can be seen that, for a light power of 250 mW, although a light profile with a smaller FWHM will lead to a larger spin current in the absorption unsaturation area, this effect is overwhelmed by the more energy wasted in the absorption saturation area. As a result, the light profile with a smaller value of *σ* (i.e., *σ* = 0.2 mm) generates a smaller value of PISHE than that with larger *σ* (i.e., *σ* = 0.3 or 0.5 mm).

**Fig. 4:**
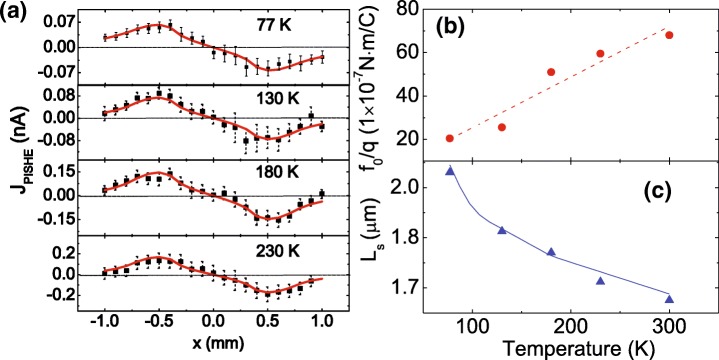
Temperature dependence of the PISHE current, spin transverse force, and electron diffusion length of the GaAs/AlGaAs 2DEG. **a** Experimental and modeling results of the PISHE current as a function of the light spot locations measured at different temperatures. The solid squares are the experimental data, and the solid lines are the fitting results. **b**, **c** Spin transverse force and electron diffusion length as a function of temperature, respectively. The dashed line in **b** is the guide for the eyes, and the solid line in **c** is obtained from [42]

To investigate the dominant mechanism of the PISHE, we carry out the PISHE measurements at different temperatures. Figure 4a shows the PISHE current as a function of the light spot location measured at 77, 130, 180, and 230 K. The laser spot has a Gaussian profile with *r*_0_ = 0.7 mm and *σ* = 0.2 mm, and the power is 250 mW. The squares indicate the experimental data, and the solid lines are the fitting results using Eqs. (2) and (4). It can be seen that the experimental data are all well-fitted by the model at all temperatures. By the fitting, we can obtain the spin transverse force *f*_0_/*q*, which is shown in Fig. 4b, and the diffusion length of electrons *A*·*L*_*s*_ at different temperatures. The dashed line in Fig. 4b is the guide for the eyes. To determine the value of the parameter *A*, we should compare the temperature dependence of *A*·*L*_*s*_ to the previous results of temperature dependence of electron diffusion length *L*_*s*_. By fitting our value of *A*·*L*_*s*_ to the value of *L*_*s*_ obtained in [42], we can determine the constant *A* to be 1.65 × 10^2^. The very good agreement of our results with previous results shown in Fig. 4c verifies our method. It can be seen that the electron diffusion length decreases with increasing temperatures, which can be mainly attributed to the enhancement of carrier scattering by phonons [43].

Surprisingly, the spin transverse force *f*_0_/*q* of the 2DEG increases monotonously with the increasing temperature, which shows an opposite variation trend on temperature for the PISHE observed in Au/InP hybrid structures [44]. This unexpected phenomenon may be related to the mechanism of PISHE. There are two mechanisms for the PISHE in semiconductor 2DEG, i.e., intrinsic and extrinsic mechanisms. The former one mainly arises from the band structure, and the latter one originates from the asymmetries in scattering for up and down spins due to the SOC effect in impurities [7, 16]. For a semiconductor 2DEG of *C*_2*v*_ point group symmetry, the spin transverse force induced by intrinsic mechanism can be expressed as $f_{0}=\frac {4m^{*2}\tau _{s}D}{\hbar ^{2}}\left (\alpha ^{2}+\beta ^{2}\right)$ [20, 40], where $\hbar $ is the reduced Planck constant, *τ*_*s*_ is the spin relaxation time, *D* is the spin diffusion coefficient, and *α* (or *β*) is the Rashba (or Dresselhaus) constant which is proportional to the strength of Rashba (or Dresselhaus) SOC. For a GaAs/AlGaAs 2DEG, the spin relaxation time *τ*_*s*_ is proportional to *T*^−1^ [45]. Here, *T* represents temperature. For the modulation doped 2DEG, the strength of Rashba SOC is much larger than that of Dresselhaus (see the following discussion); as a result, the Rashba constant *α* is much larger than Dresselhaus constant *β*. The spin diffusion coefficient *D* is proportional to *T*^−2^ [46, 47]. The temperature dependence of *α* can be expressed as *a*+*bT*, where *a* and *b* are constants, and *a* is about two orders larger than *b* [48]. Thus, taking into account the temperature dependence of *τ*_*s*_, *D*, and *α*, we have *f*_0_∝*T*^−3^, which suggests that the spin transverse force induced by the intrinsic mechanism should decrease with increasing temperatures. For the extrinsic mechanism, the spin transverse force is dependent on the concentration of ionized impurity, especially for extrinsic side-jump scattering [49, 50]. Since there is background doping in our sample, and the impurity ionization increases with increasing temperature, stronger asymmetry scattering for spin-up and spin-down electrons happens as temperature increases, leading to larger spin transverse force with increasing temperature. Given that the spin transverse force *f*_0_ observed in our experiment increases with increasing temperature, it can be inferred that the PISHE is dominated by the extrinsic mechanism, in which the impurities are mainly introduced by the background doping during the growth process.

**Fig. 5:**
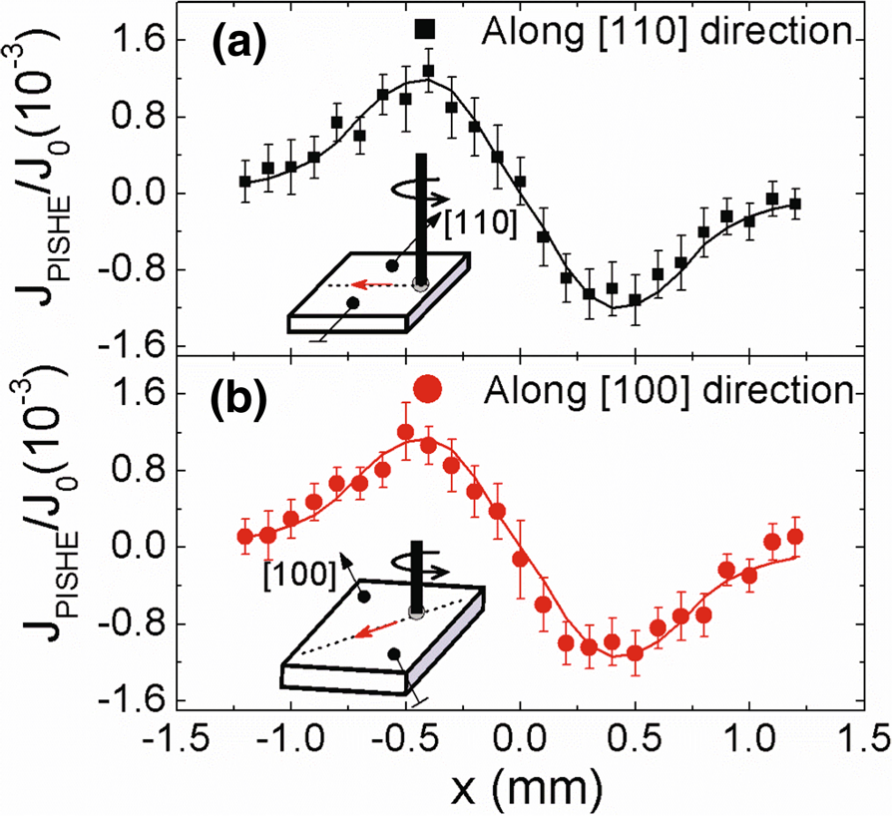
The normalized PISHE current collected along different crystal orientations. The PISHE current is normalized by the photocurrent under a bias of 0.3 V. The solid symbols are the experimental data, and the solid lines are the theoretical fitting results. The insets show the schematic diagrams for the corresponding measurement geometries

To further confirm that the PISHE is indeed dominated by the extrinsic mechanism, we measure the spatial distribution of the PISHE current collected along different crystal directions. Figure 5a, b shows the spatial distribution of the PISHE current collected along [110] and [100] crystal directions, respectively. To eliminate the influence of the carrier mobility and the carrier density in different crystal directions, we normalize the PISHE current by the corresponding photocurrent *J*_0_ under a bias of 0.3 V when the contacts are along [110] and [100] directions, respectively. The measurements are carried out at room temperature under a radiation of power 60 mW. The light spot radius *r*_0_ is 1.0 mm, and *σ* is 0.3 mm. The symbols indicate the experimental data, and the solid lines are the fitting results according to Eqs. (2) and (4). It can be seen that there is no marked difference between the normalized PISHE current collected along [110] and [100] crystal directions.

**Fig. 6:**
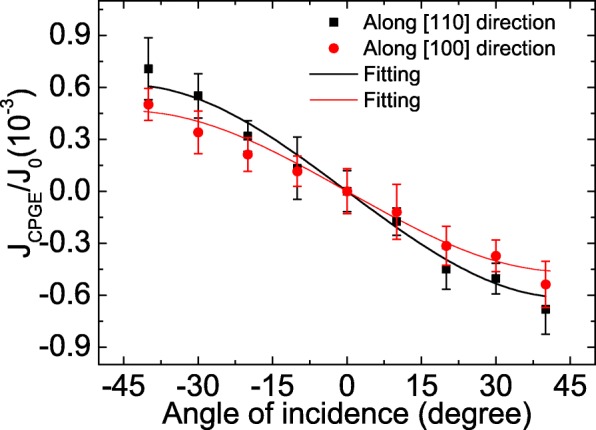
Incident angle dependence of the normalized CPGE current collected along different crystal orientations. The CPGE current is normalized by the photocurrent under a bias of 0.3 V. The solid symbols are the experimental data, and the lines are the fitting results according to Eq. (5)

For intrinsic mechanism, the PISHE current along a certain crystal direction is related to the spin splitting at that direction. To get the knowledge of the anisotropy spin splitting in the GaAs/AlGaAs 2DEG, we measure the crystal orientation dependence of the CPGE current at room temperature, i.e., we measured the CPGE current when the two contacts are along [110] (or [100]) direction and the incident plane of light lies in [1$\bar {1}$0] (or [010]) direction, of which the measurement results are shown by squares (or by circles) in Fig. 6. It is worth mentioning that when measuring the CPGE current, the light spot is located at the midpoint of the connection of the two contacts, where the PISHE is zero according to [20]. The CPGE current is also normalized by the corresponding photocurrent under a bias of 0.3 V to eliminate the influence of the carrier mobility and the carrier density in different crystal directions [51]. Then, we use the following equation to fit the normalized angle-dependent CPGE current to obtain the relative SOC strength along different crystal directions [21, 27]:

5 $$ \begin{aligned} J^{\lambda}/J_{0}=\frac{A_{\lambda}\sin \theta \cos^{2} \theta}{n\left[\cos\theta+\left(n^{2}-\sin^{2} \theta\right)^{1/2}\right]\left[n^{2}\cos \theta +\left(n^{2}-\sin^{2}\theta\right)^{1/2}\right]}. \end{aligned}  $$

Here, *θ* is the angle of incidence, *n* is the refractive index of GaAs, and *A*_*λ*_ is a constant proportional to the SOC constant. The fitting results are shown by the solid lines in Fig. 6. When the incident plane of light lies in [1$\bar {1}$0] direction and the CPGE current is collected along [110] direction, the corresponding *A* parameter, denoted as *A*_[110]_, is proportional to the sum of Rashba and Dresselhaus SOC, i.e., *A*_[110]_∝*α*+*β* [51–53]. When the incident plane of light lies in [010] direction and the CPGE current is collected along [100] direction, the corresponding *A* parameter, denoted as *A*_[100]_, is proportional to the Rashba SOC, i.e., *A*_[100]_∝*α* [51–53]. Thus, by the ratio of *A*_[110]_/*A*_[100]_, we can get the relative ratio of Rashba to Dresselhaus SOC, i.e., $\beta /\alpha =\frac {A_{[110]}}{A_{[100]}}-1$ = 0.32, which indicates that the spin splitting in the GaAs/AlGaAs 2DEG has crystal anisotropy [21]. Therefore, the intrinsic contribution to the PISHE should be sensitive to the crystal axis [16]. Specifically speaking, according to Eqs. (2) and (4), when the contacts are along [110] (or [100]) direction, the measured PISHE current is dominated by the inverse spin Hall current flowing nearly parallel to [110] (or [100]) direction since the PISHE current is a vortex current. If the intrinsic mechanism plays a dominant role in the 2DEG, the PISHE current collected along these two directions should be different. However, no marked difference is observed, which suggests that the extrinsic mechanism is dominant in the GaAs/AlGaAs 2DEG.

## Conclusions

In conclusion, the PISHE current in a GaAs/AlGaAs 2DEG has been investigated in a temperature range of 77 to 300 K. The spin transverse force has been determined by fitting the PISHE current to a theoretical model. The dependence of the PISHE on the light power and on the light spot profiles has been investigated, which shows a good agreement with the theoretical model. The evolution of the PISHE current with temperature suggests that the PISHE is dominated by the extrinsic mechanism, which is further confirmed by the weak dependence of the PISHE current on the crystal orientation of the sample.

## Abbreviations

2DEG: Two-dimensional electron gas
CPGE: Circular photogalvanic effect
EMF: Circular electromotive force
FWHM: Full width at half maximum
ISHE: Inverse spin Hall effect
MBE: Molecular beam epitaxy
PISHE: Photo-induced inverse spin Hall effect
SHE: Spin Hall effect
SOC: Spin-orbit coupling
